# Editorial for the Special Issue on Particles Separation in Microfluidic Devices

**DOI:** 10.3390/mi11060602

**Published:** 2020-06-22

**Authors:** Naotomo Tottori, Takasi Nisisako

**Affiliations:** 1 Department of Mechanical Engineering, Faculty of Engineering, Kyushu University, W4-729, 744, Motooka, Nishi-ku, Fukuoka 819-0395, Japan; tottori@mech.kyushu-u.ac.jp; 2 Institute of Innovative Research, Tokyo Institute of Technology, R2-9, 4259 Nagatsuta-cho, Midori-ku, Yokohama, Kanagawa 226-8503, Japan

The separation and sorting of micro- and nano-sized particles is an important step in chemical, biological, and medical analyses. In the past two decades, micro- and nanofluidic platforms have been increasingly applied for the separation, fractionation, sorting, and purification of all classes of particles based on their physical and chemical properties because of their advantages of minimal consumption of sample and reagent, ease of use, and enabling of the integration of multicomponent for comprehensive analysis. The separation techniques using micro- and nanofluidic devices are classified into passive methods using geometries and hydrodynamic effects at micro/nanoscale, and active methods using external fields such as electric, magnetic, optical, and acoustic forces.

This Special Issue collects some state-of-the-art developments in active and passive microfluidic separation, isolation, and manipulation for a wide range of particles. In this Special Issue, 11 research papers, and two review articles are published. Five papers [1,2,3,4,5] and a review article [6] present (1) passive microfluidic techniques using inertial focusing [1,6], deterministic lateral displacement (DLD) [2,3], and hydrodynamic methods [4,5]. The remaining papers [7,8,9,10,11,12] and a review article [13] cover (2) active microfluidic techniques using electric [7,8,9], acoustic [10], magnetic [11,12], and optical forces [13].

(1) Passive microfluidic technique: Bogseth et al. proposed a co-flow inertial microfluidic device that is tunable in multiple ways for adaptation to different application requirements [1]. They evaluated flow rate, flow rate ratio, and output resistance ratio to flexibly tune the cutoff size of the device and separation performance even after the devices are fabricated. Kottmeier et al. experimentally observed an asymmetric flow field pattern caused by vortices behind DLD mircopost at high Reynolds number (Re > 1) using microparticle image velocimetry and compared this experimental result with CFD simulations [2]. Jiao et al. reported a numerical simulation of the motion of red blood cells (RBCs) flowing through DLD devices with different pillar shapes and gap configurations [3]. Eluru et al. proposed a microfluidic in-flow decantation technique that enables continuous separation of particles from fluid [4]. They achieved clog-free separation during the operation for at least an hour and could obtain purities close to 100% and yields as high as 14%. Yanai et al. demonstrated a new hydrodynamic mechanism of microparticle separation using dual-depth, lattice-patterned asymmetric microchannel networks [5]. By precisely observing the motion of model particles in the microchannel, they revealed that the 3D laminar flow profile affects the size-selective particle separation. They also demonstrated that the input position of particles in both x and z directions could improve the separation performance significantly. In addition to these research articles for passive techniques, Volpe et al. wrote a comprehensive review of microfluidic particles sorting using inertial focusing and laminar vortex technology [6].

(2) Active microfluidic technique: Krishna et al. presented an experimentally validated mathematical model of a microfluidic device with nozzle-shaped electrode configuration for dielectrophoretic 3D-focusing of particles [7]. They investigated the effect of operating/geometric parameters on the 3D-focusing efficiency of the device through the proposed mathematical model. Alnaimat et al. conceptualized and mathematically modeled a dielectrophoretic microfluidic device with two sets of interdigitated transducer vertical electrodes for separation of a binary heterogeneous mixture of particles based on size [8]. The proposed model is used for a parametric study to investigate the effect of parameters on the performance of the microfluidic device. Gudagunti et al. used negative dielectrophoresis (DEP) spectroscopy as an effective transduction mechanism of a biosensor to accurately detect single nucleotide polymorphism (SNP) in a short DNA strand [9]. Clark et al. demonstrated real-time monitoring of voltage measurements and immediate, corresponding adjustments to acoustic trapping frequency to improve their acoustic differential extraction [10]. Kang et al. introduced positive and negative methods for isolating circulating tumor cells (CTCs) by lateral magnetophoresis [11]. They compared the CTCs recovery rates, WBC depletion rates, and CTC purities between the positive and negative methods to discuss their strengths and weaknesses points for CTC-based diagnostics, prognostics, and therapeutics for cancer. Sobecki et al. reported numerical simulation of the dynamics of a paramagnetic elliptical particle in a low Reynolds number Poiseuille flow in a curved channel and under a uniform magnetic field [12]. In addition to these research articles for active techniques, Zhang et al. presented a comprehensive review of the latest progress in fiber optofluidics (FOF) based on two major opto-physical effects, namely optical force and the photothermal effect, in manipulation and sensing applications [13].

We would like to thank all authors for submitting their papers to this Special Issue. We would also like to acknowledge all the reviewers for dedicating their time and timely reviews to improve the quality of this Special Issue.
